# Grading detection of “Red Fuji” apple in Luochuan based on machine vision and near-infrared spectroscopy

**DOI:** 10.1371/journal.pone.0271352

**Published:** 2022-08-04

**Authors:** Jin Wang, Yujia Huo, Yutong Wang, Haoyu Zhao, Kai Li, Li Liu, Yinggang Shi

**Affiliations:** College of Mechanical and Electronic Engineering, Northwest A&F University, Yangling, Shaanxi, 712100, China; Accra Technical University, GHANA

## Abstract

A quality detection system for the “Red Fuji” apple in Luochuan was designed for automatic grading. According to the Chinese national standard, the grading principles of apple appearance quality and Brix detection were determined. Based on machine vision and image processing, the classifier models of apple defect, contour, and size were constructed. And then, the grading thresholds were set to detect the defective pixel ratio *t*, aspect ratio λ, and the cross-sectional diameter W_p_ in the image of the apple. Spectral information of apples in the wavelength range of 400 nm~1000 nm was collected and the multiple scattering correction (MSC) and standard normal variable (SNV) transformation methods were used to preprocess spectral reflectance data. The competitive adaptive reweighted sampling (CARS) algorithm and the successive projections algorithm (SPA) were used to extract characteristic wavelength points containing Brix information, and the CARS-PLS (partial least squares) algorithm was used to establish a Brix prediction model. Apple defect, contour, size, and Brix were combined as grading indicators. The apple quality online grading detection platform was built, and apple’s comprehensive grading detection algorithm and upper computer software were designed. The experiments showed that the average accuracy of apple defect, contour, and size grading detection was 96.67%, 95.00%, and 94.67% respectively, and the correlation coefficient R_p_ of the Brix prediction set was 0.9469. The total accuracy of apple defect, contour, size, and Brix grading was 96.67%, indicating that the detection system designed in this paper is feasible to classify “Red Fuji” apple in Luochuan.

## 1 Introduction

Grading for sales is strongly needed to commercialize agricultural products and boost economic benefits [1]. Automated apple grading can reduce manual work intensity and improve the repeatability and accuracy of results. The external quality parameters for assessing apple grades include surface defects, color, texture, size, and shape. The internal quality parameters for assessing apple grades include degrees Brix, acidity, vitamins, water content, soluble solids, internal quality defects, etc [2,3]. Based on these indicators, many experts and scholars have carried out relevant research on automatic apple grading. Among them, there are a relatively large number of studies on automatic apple grading algorithms based on machine vision [4–6]. Additionally, in terms of injury-free detection technology for agricultural products, spectroscopy technology has undergone rapid development [7–9]. Zhao Miao et al. developed a robotic system for the automatic detection and classification of internal quality attributes of apples using near-infrared spectroscopy [10]; Liu Penghui et al. used machine vision and spectroscopy for the non-destructive detection of apple crispness with accurate and reliable results [11]. Tan Wenyi et al. used hyperspectral imaging to propose an accurate algorithm for apple abrasion identification, which provided a new method for non-destructive detection [12]. Keresztes et al. used shortwave infrared hyperspectral imaging combined with calibration and glare correction techniques, on a real-time pixel basis for contusion detection of early apples [13].

In 2020, China produced 44.066 million tons of apples, exceeding the global average production by more than 50%, among which, approximately 1812106.13 acres of apples were planted in Shaanxi Province, producing 11.8521 million tons [14], showing great demand for automated apple grading machines. To carry out automatic grading of apples comprehensively and accurately, this study designed a set of grading detection algorithms for Red Fuji apple in Luochuan, mainly including four single quality detection algorithms and a comprehensive grading algorithm. Firstly, three classifier models of defect, contour, and size were designed by using machine vision, and the defect, contour, and size of the apple’s appearance were detected successively. Secondly, near-infrared spectroscopy was used to collect the spectral information of apples. And CARS-PLS algorithm was adopted to predict the sugar content of apples. Finally, according to the National Standard of the People’s Republic of China for grading fresh apples, combined with the defect, contour, size, and Brix of the detected apple, the comprehensive grading algorithm of Red Fuji apples in Luochuan was designed. With the algorithm, upper computer software was designed to display the grading detection results in real-time. An online grading detection platform for apple quality was built to verify the effectiveness and accuracy of the grading algorithm. Experimental results show that the accuracy of the designed algorithm is 96.67%, which met the requirements of automatic grading of the Red Fuji apple in Luochuan.

## 2 Experimental materials

**Fig 1:**
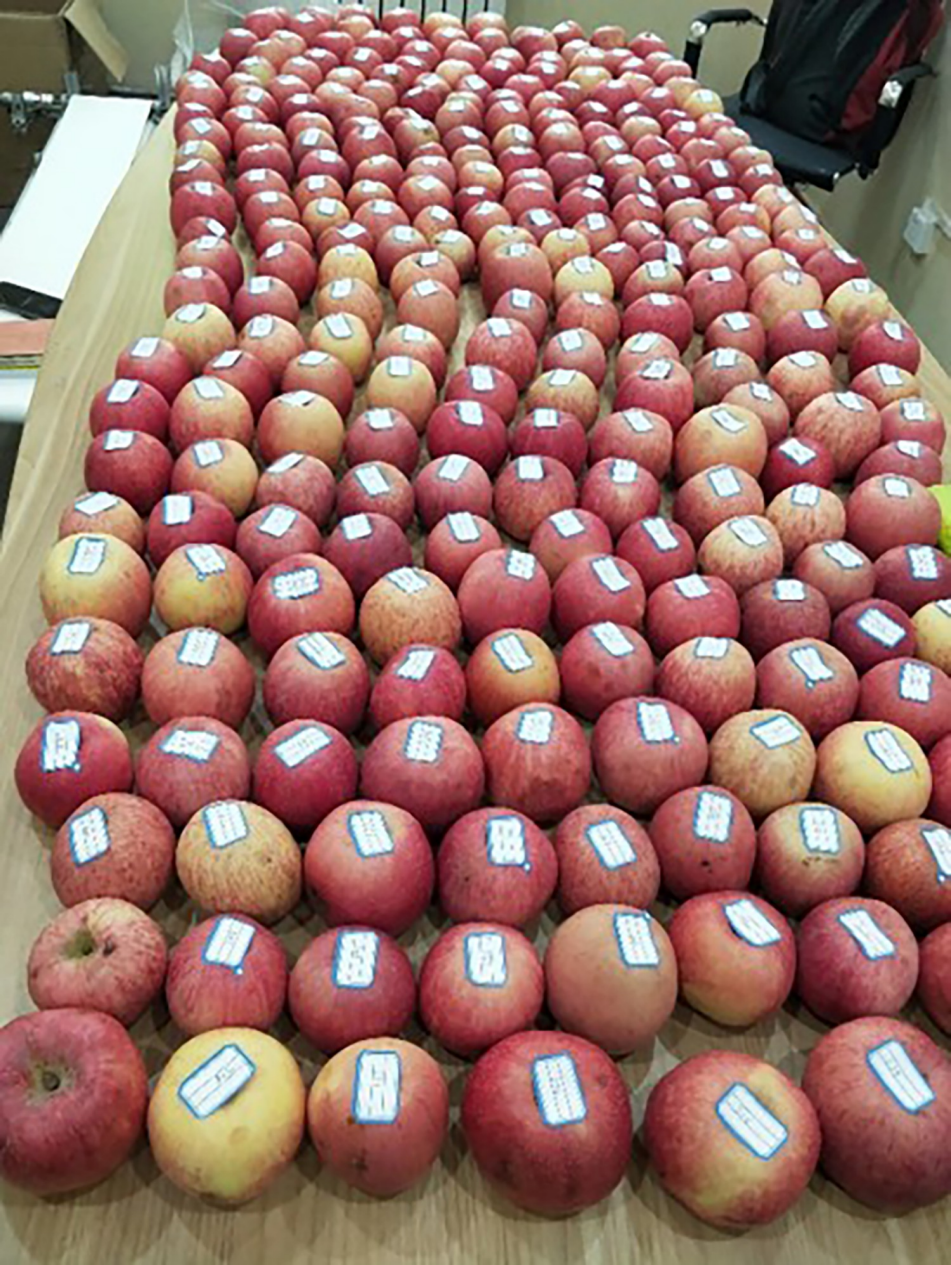
Red Fuji apple appearance.

The apples selected in this study are Red Fuji varieties in Luochuan, Shaanxi Province, which were purchased from Shenggu Industrial Co Ltd in Yan’an, Shaanxi Province. Before experiments, each sample was numbered, and the serial number label was attached to the bottom of the sample. Their appearance is shown in Fig 1.

## 3 Principle of apple quality detection classifier

### 3.1 Principle of apple appearance quality grading detection

According to the National Standard of the People’s Republic of China for grading fresh apples [15], apples are classified according to their size characteristics based on their cross-sectional diameters, i.e., the diameter at the largest point in the cross-section, as follows: the apple with a cross-sectional diameter >7 cm is graded large (L), the apple with a cross-sectional diameter >6.5 cm and <7 cm is graded medium (M), and the apple with a cross-sectional diameter <6.5 cm is graded small (S).

Similarly, the national standard for apple grading is based on varietal characteristics, shape characteristics (fruit shape index), color, red coloring rate, pests, or other damages, and the test indicators are as follows:

Excellent fruit: uniform in appearance, round and free of ribs, square, round or nearly round, without skew, with a fruit shape index of 0.85 or more; evenly distributed color, high brightness; smooth and delicate, evenly distributed texture, not rough, without damages, cracks or scars.

First-class fruit: undifferentiated in appearance, nearly round or oblate, slightly deformed but not more than 30% of the total, with a fruit shape index of 0.8 to 0.85; ripe or slightly underripe, evenly distributed color, evenly distributed texture, not rough; without damages, cracks or scars.

Second-class fruit: basic fruit shape, subrounded, slightly deformed, with defects in fruit shape but still having basic features, no malformation, with a fruit shape index of 0.75 to 0.8; slightly under- or overripe, slightly rough; slightly damaged, no cracks, slight scars on the skin, but unaffected fruit merchantability.

Substandard fruit: variable appearance in shapes, oval-shaped fruit with large deformation, with a fruit shape index of 0.75 or less; less evenly distributed texture, slightly under- or overripe, slightly rough; with damage cracks and obvious scars on the skin.

Most of the factors affecting apple grading are sensory factors. According to the National Standard for grading fresh apples, the defect, contour, and size of the Red Fuji apple in Luochuan were selected as external quality detection indexes in this study. In general, apple surface defects are usually judged by the human eye. Contour is generally evaluated by fruit shape coefficient, which is usually the ratio of transverse diameter to longitudinal diameter. The closer the fruit shape coefficient is to 1, the rounder the apple appearance. And the apple size is generally evaluated by measuring the maximum cross diameter with a vernier caliper. The above measurement methods of apple defect, contour, and size are physical detection methods, which need to be completed manually. They are not efficient and automatic enough to meet the requirements of real-time apple grading detection. Therefore, this study proposed methods of processing apple images by machine vision to complete the real-time detection of the apple defect area, aspect ratio, and maximum transverse diameter. Then, each external quality was graded according to the national standards.

### 3.2 Principle and method of apple Brix detection

Currently, China’s national standard for apple internal quality only gives a reference index of 7% or more for fruit hardness and 13% or more for soluble solids in mature Fuji apples. The unclear national standard for the internal quality of apples has resulted in a lack of scientific, systematic, and operable indicators of grading evaluation based on this standard.

Customers pay attention to taste when buying apples. The most important factor affecting the taste of apples is their sugar-acid ratio, of which the apple Brix value plays a leading role. Current chemical methods for measuring the apple Brix value include 3, 5-dinitrosalicylic acid colorimetric method, anthrone colorimetric method, Fehling reagent thermal titration, and so on [16]. All the above determination methods need to use acid to hydrolyze disaccharides and polysaccharides into reductive monosaccharides and use the reductive sugar determination method to measure the sugar content of apples. In addition, gas chromatography and high-performance liquid chromatography can not only measure sugar content but also determine the composition of sugar. Generally, recovery and precision experiments are used to evaluate the above chemical methods, and the accuracy of measured sugar content is evaluated by calculating the total sugar recovery, standard deviation, and coefficient of variation. The more the total sugar recovery is close to 100%, the smaller the standard deviation and coefficient of variation are, and the more accurate and reliable the measured sugar content is. These methods, however, generally require the apple to be mashed and macerated into a solution for measurement, which causes damage to the fruit and takes a long time with low efficiency, thus they are not suitable for non-destructive apple grading.

When near-infrared light is directed at a suitable angle to the apple, the chemical groups within the apple absorb the spectral energy and produce a diffuse reflection on the surface, the intensity of which varies according to the degree of light absorption within the apple. Apple sugars contain mainly C-H and O-H groups, which are located at different energy levels and absorb different energies from different wavelengths of the spectrum. Additionally, these are selective in their absorption of light, with the spectrum being absorbed only at specific frequencies. Therefore, apple sugars can produce characteristic absorption of near-infrared (NIR) radiation, and the difference in absorption at different wavelengths also affects the reflected energy of diffuse reflection [17]. Therefore, in this study, near-infrared spectroscopy technology was used to collect the spectral reflectance data from apple samples within the 400 nm~1000 nm wavelength region. And three chemometrics methods, PLS algorithm, CARS-PLS algorithm, and SPA-PLS algorithm, were used to build the mapping relationship between the spectral reflectance data and the apple Brix. Three methods were compared and analyzed to select the optimal method to measure the apple Brix.

#### 3.2.1 PLS

PLS algorithm is a linear regression algorithm, not affected by the dimension, and suitable for complex near-infrared spectral analysis. PLS algorithm can effectively eliminate the collinearity of wavelengths. If the model contains a large number of useless information variables, the prediction ability of PLS will also be affected [18]. In this study, the PLS algorithm was used to establish a prediction model between the spectral data of the whole band and apple Brix.

#### 3.2.2 CARS-PLS

CARS algorithm is a feature variable selection method combining Monte Carlo sampling and PLS model regression coefficient, imitating the principle of "survival of the fittest" in Darwin’s theory. Each time, wavelength points with the large absolute weight of regression coefficients in the PLS model were retained as new subsets through adaptive weighted sampling, and then the PLS model was established based on the new subsets. After multiple iterative calculations, the subset with the minimum root mean square error of cross-validation (RMSECV) was selected as characteristic variables [19]. In this study, characteristic variables selected by the CARS algorithm and actual Brix of apples were used to establish a PLS linear regression model.

#### 3.2.3 SPA-PLS

SPA is an algorithm that uses a forward cycle to screen variables, and the extracted feature bands have low collinearity, which effectively avoids information overlap and reduces the amount of calculation [20]. The SPA algorithm was also adopted to screen characteristic variables to optimize the effect of the PLS algorithm on predicting apple Brix in this study.

## 4 Classifier construction for apple appearance quality detection

### 4.1 Apple image capture

**Fig 2:**
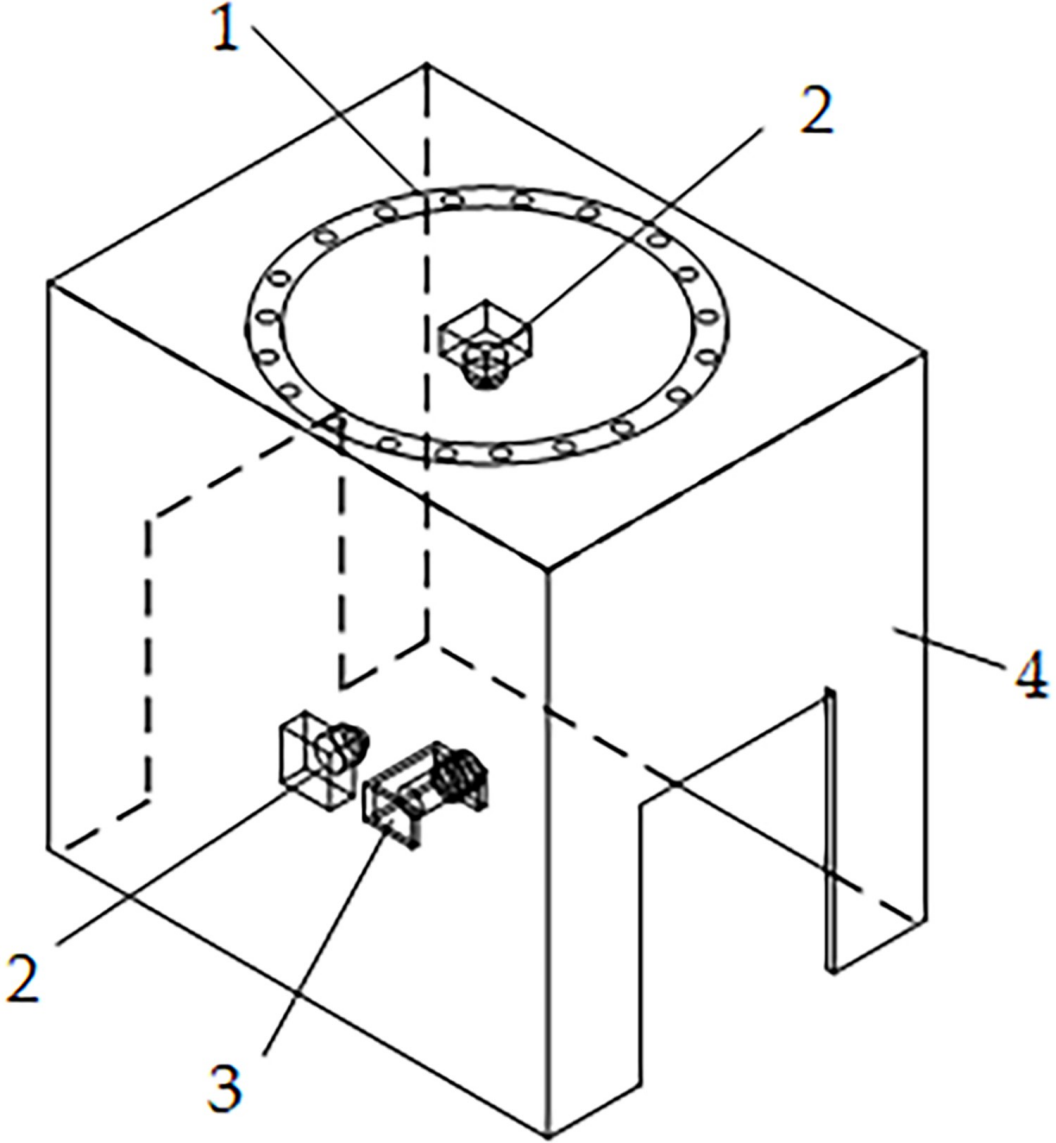
Sketch of the structure of the vision-based detection device. 1-ring light source 2-camera 3-photoelectric switch 4-dark box housing.

The vision-based apple appearance detection device, shown in Fig 2, consists of a ring light source (2835–120, Transcend, Shenzhen), two cameras (HF899, Jereh Microcom, Shenzhen), a photoelectric switch (E3F-DS30C4, Hutron, Shanghai), and a dark box housing made of polymethyl methacrylate (PMMA). Among them, the ring light source is adhered to the upper panel of the housing to provide a light environment, and the top camera is mounted right in the middle of the ring light source and connected to the universal serial bus (USB) port of an external personal computer (PC) (HUAWEI MateBook 14, Huawei Technologies Co Ltd, Shenzhen) through a predetermined external hole. The side-mounted photoelectric switch is used to detect the passage of fruits on the conveyor belt, and two cameras take pictures when apples are passed.

### 4.2 Image preprocessing

**Fig 3:**
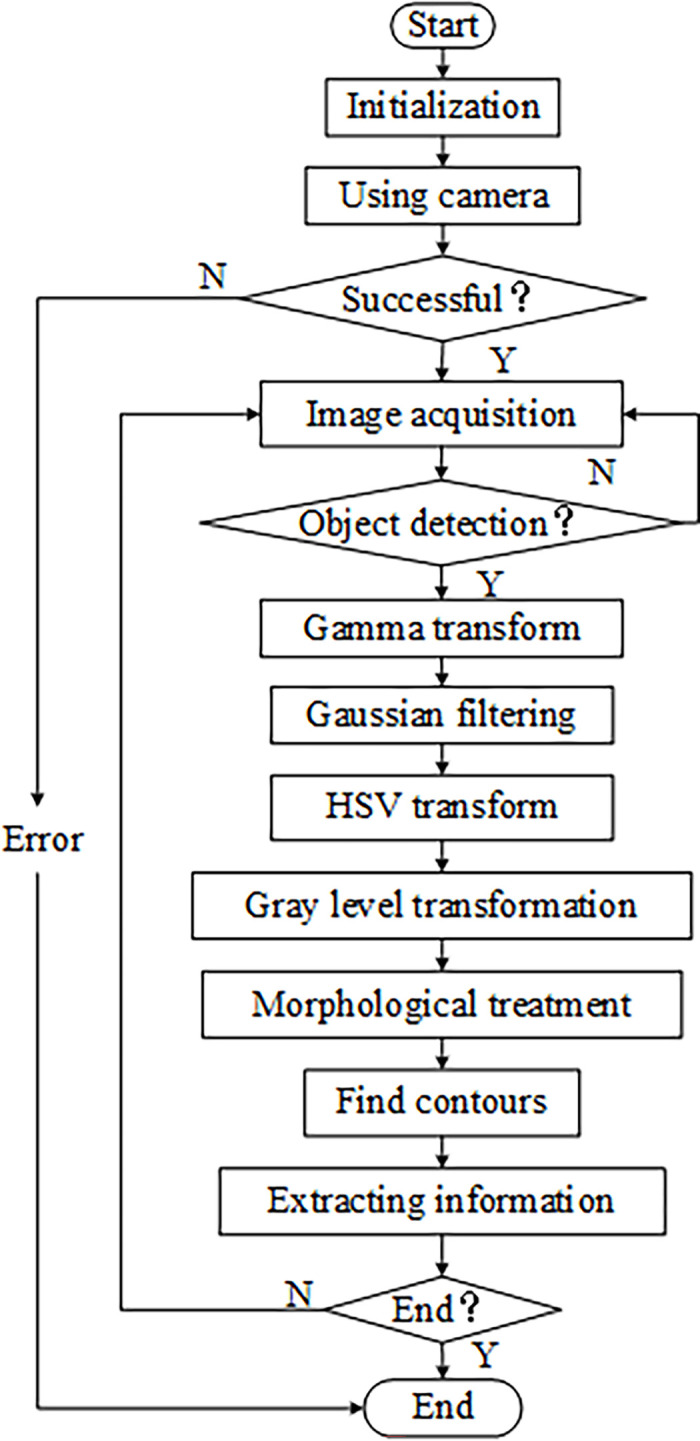
Image processing flow.

Acquiring and processing images of Red Fuji apples in Luochuan, Shaanxi Province, and extracting and analyzing surface feature data such as the defects, size, and shape of the apples are types of image preprocessing, and this algorithm flow is shown in Fig 3. First, the camera was used to directly acquire the original image of the apple; then, preprocessing methods such as image enhancement, filtering, and morphological processing were used to eliminate background interference and improve the accuracy of segmentation; next, the apple surface color was selected as the distinguishing feature for segmentation of the apple fruit from the background.

**Fig 4:**
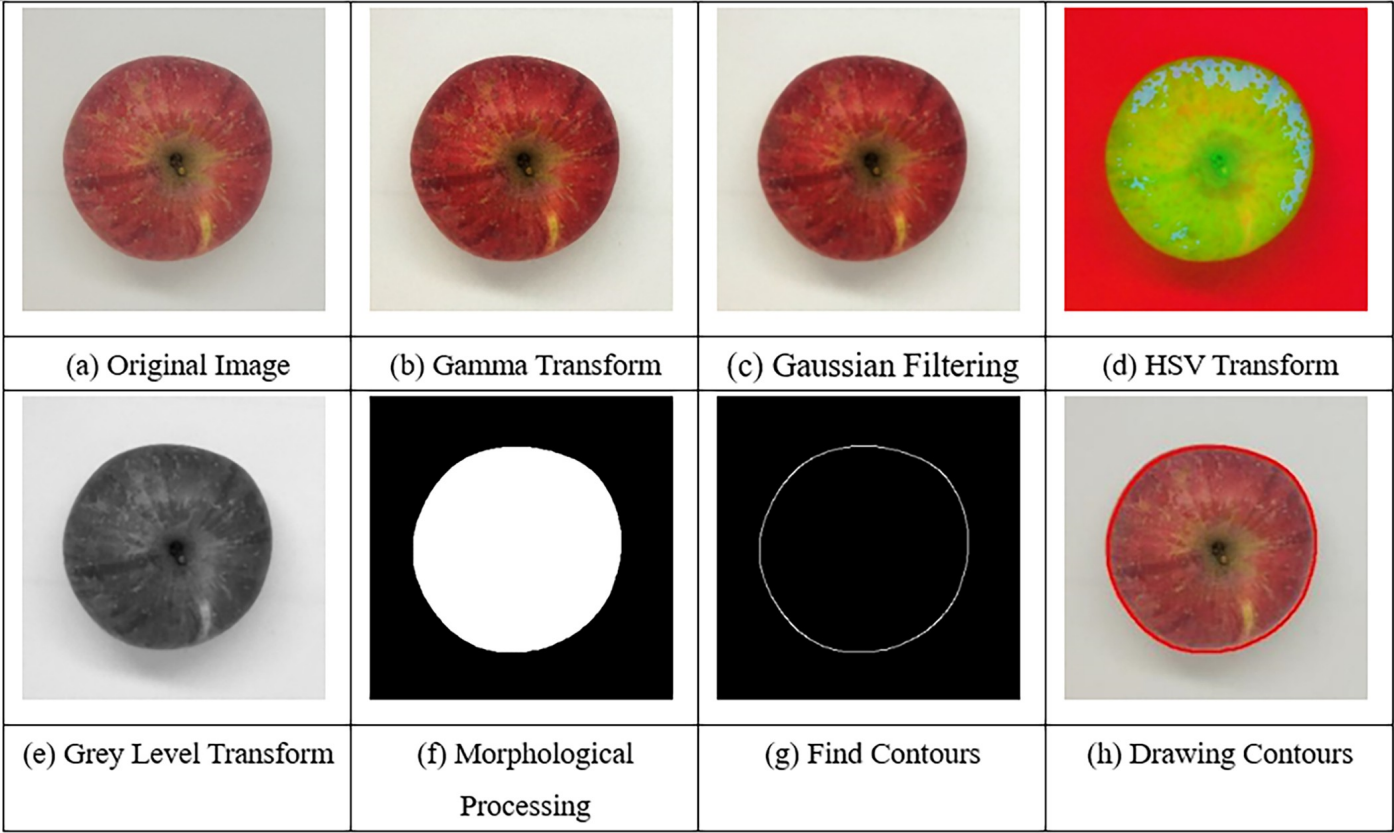
Image preprocessing process. (a) Original image (b) Gamma transform (c) Gaussian filtering (d) HSV transform (e) Grey level transform (f) Morphological processing (g) Find contours (h) Drawing contours.

The apple image preprocessing process is shown in Fig 4. The grey level of the original image shown in Fig 4(A) is corrected using standard color plates and the contrast of the image is enhanced using the gamma transform [21], as shown in Fig 4(B). Then, the image noise is suppressed by using Gaussian filtering [22], preserving important information such as image contours and edges, as shown in Fig 4(C). Next, the red, green, and blue (RGB) image is transformed into the hue, saturation, and value (HSV) image shown in Fig 4(D), and the grey level transformed and threshold segmentation [23] were performed to distinguish the white background from the fruit area, as shown in Fig 4(E). Morphological processing was used to eliminate the tiny discrete closed area and boundary interference [24] in the white background, as shown in Fig 4(F). The interior of the highlighted area, where the fruit is located, may also be mistaken for the black area due to the fruit tip as well as surface reflections. Multiple contours can be found by finding the boundary pixel mutation points between the highlighted area and the black area. The largest contour is extracted as the apple contour, as shown in Fig 4(G). The pixel points at the edge of the contour are selected to draw the apple contour, as shown in Fig 4(H).

### 4.3 Defect determination

Apple surface defect types mainly include rot, scars, wormholes, and crush injuries. The defective area can be extracted by grey level transformation and threshold segmentation of the preprocessed apple image. However, the national standard does not give a clear definition. To obtain the size of the area threshold for defect determination, based on the sensory judgment and the market research, the minimum diameter of the defective area for the apple with a 70 mm medium diameter is 3 mm, and the critical defect ratio for the apple surface defect is defined *t*_*0*_

$$t_{0}=\frac{\pi \times d_{0}^{2}}{\pi \times d_{1}^{2}}\times 100\%=\frac{3^{2}}{70^{2}}=0.18\%$$ (1)

If the defective pixel ratio *t* is greater than or equal to *t*_*0*_, the apple is evaluated to be defective; otherwise, it is evaluated to be normal, and the classifier for detecting apple defects is

$$t=\{\begin{array}{l} 0\sim 0.0018,\;Normal\;apples\\ \geq 0.0018,\;Defective\;apples \end{array}$$ (2)

**Fig 5:**
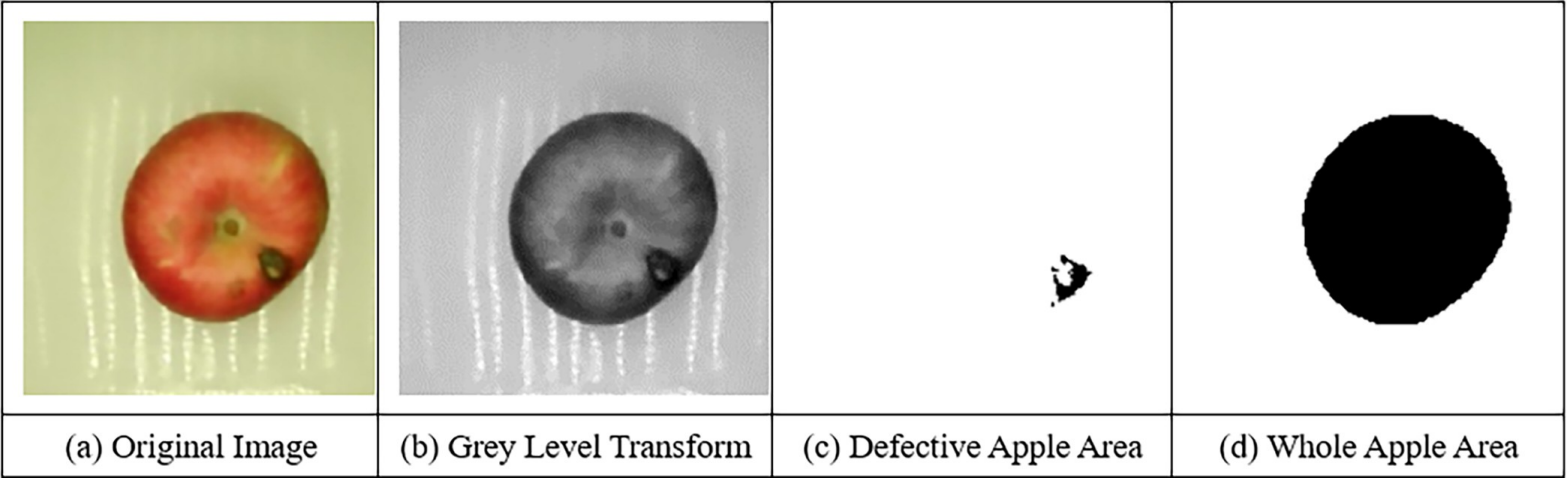
Process of extracting defective area of the apple. (a) Original image (b) Grey level transform (c) Defective apple area (d) Whole apple area.

Fig 5 shows the image processing process of defect determination. Forty apples were collected, half of them were defective and the other half were not, and the experiment was conducted in triplicate with the use of the defect classifier. The results are shown in Table 1, and the average accuracy of the defect classifier was 96.67%, indicating that this model can be used in the grading detection of apple defects.

**Table 1:** Results of detection for apple defects.

Testing index	Detection number	First	Second	Third
Defective apples	20	20	20	18
Non-defective apples	20	20	19	19
Error		0	1	3
Accuracy		100%	97.5%	92.5%

### 4.4 Shape determination

**Fig 6:**
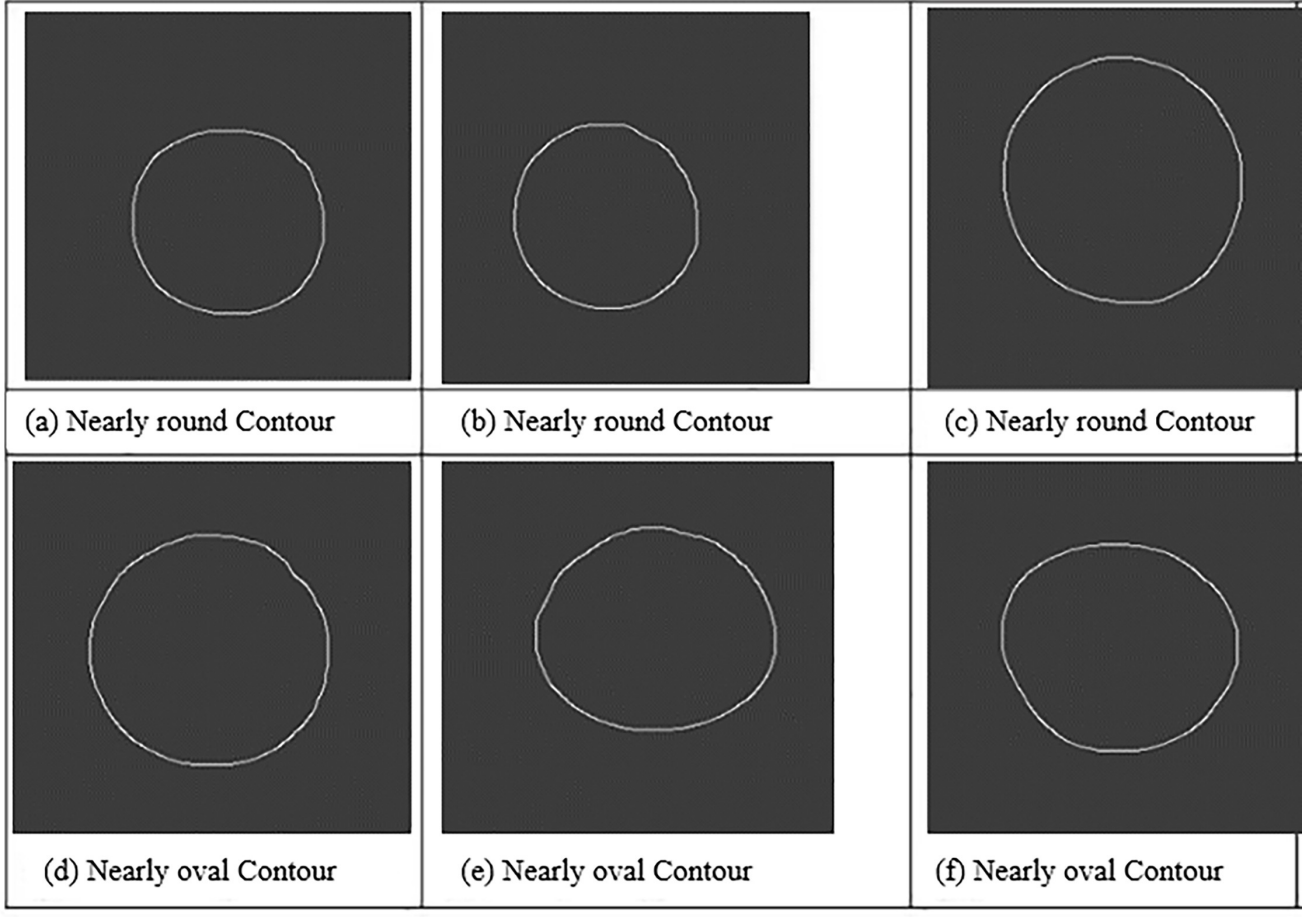
Typical contours of apples. (a)~(c) Nearly round contour (d)~(f) Nearly oval contour.

From the top view, the shape of the apple was roughly classified into round and oval, as shown in Fig 6, where (a) to (c) are the contours of nearly round apples and (d) to (f) are those of nearly oval apples. Customers usually have a higher preference for round apples than for misshapen apples. According to the national standard for grading apples and the consumption habits, the apple images were preprocessed to extract the maximum contours and calculate the maximum distance ratio between their horizontal and vertical directions, that is, the aspect ratio *λ* [25], to construct the basic parameters of the apple contour classifier. The near round contours in Fig 6(A) to 6(C) have aspect ratios *λ* ranging from 0.98 to 1.05, while the nearly oval contours in Fig 6(D) to 6(F) have aspect ratios *λ* greater than or equal to 1.05 or less than 0.98. The apple aspect ratio *λ* can be used to evaluate the apple shape and judge its general contour. The classifier to detect the apple contour is

$$\lambda =\{\begin{array}{l} 0.98\sim 1.05,\;nearly\;round\;apples\\ \leq 0.98or\geq 1.05,\;nearly\;oval\;apples \end{array}$$ (3)

Forty apples were collected, half of them were nearly round and the other half were nearly oval, and the experiment was conducted in triplicate with the use of the shape classifier. The results are shown in Table 2, and the average accuracy of the shape classifier was 95%, indicating that the model can be used in the grading detection of apple fruit shape.

**Table 2:** Results of detection for apple shape.

Testing index	Detection number	First	Second	Third
Nearly round apples	20	19	18	19
Nearly oval apples	20	20	19	19
Error		1	3	2
Accuracy		97.5%	92.5%	95.0%

### 4.5 Size determination

The size of an apple is related to the maximum cross-sectional diameter of the fruit, so the actual cross-sectional diameter of the apple and the maximum cross-sectional diameter in the image were measured to construct a mapping relationship between the image size and the actual size of the fruit. Then, the grading system measured the maximum cross-sectional diameter in the image of the apple to be graded, and based on the mapping relationship between the actual size and the image size, the maximum cross-sectional diameter of the apple was approximated and used to distinguish among extra-large, large, medium and small apples to aid in the system to grading.

In this research, we used a vernier caliper to measure and record the maximum cross-sectional diameter W_r_ of a sample of apples, and Hu moments to measure the cross-sectional diameter W_p_ in the image. To measure the cross-sectional diameter of an apple in its image, it is important to find the center of the apple profile (*x*_c_, *y*_c_) and the boundary point of the profile (*x*_k_, *y*_k_) in the image.

Hu moments are geometric moments that characterize an image [26]. When the image function is *f*(*x*,*y*) and the resolution is *U*×*V*, the *p*+*q* order moments of the image is

$$m_{pq}=\sum_{y=1}^{V}\sum_{x=1}^{U}x^{p}y^{q}f(x,y)$$ (4)

The 0th order moments of a binary image *m*_00_ represent the area of the contour’s connected domain. Using the 0th and 1st order moments, the coordinates of the apple contour center point [27]

$$\left\{ \begin{array}{l} x_{c}=\frac{m_{10}}{m_{00}}\\ y_{c}=\frac{m_{01}}{m_{00}} \end{array}\right.$$ (5)

The radius sequence of (*x*_c_, *y*_c_) to that of the boundary point (*x*_k_, *y*_k_) in the image is

$$r_{k}={\left[{\left(x_{k}-x_{c}\right)}^{2}+{\left(y_{k}-y_{c}\right)}^{2}\right]}^{\frac{1}{2}}$$ (6)

For the extracted fruit contour in Fig 4(G), the coordinates of its geometric center can be obtained using Eq (5), and the radius sequence from the geometric center to the boundary point of the apple contour in the image can be calculated using Eq (6). By using the method of the least convex package, the maximum R_max_ in the radius sequence from (*x*_c_, *y*_c_) to (*x*_k_, *y*_k_) was filtered out [28], and the maximum cross-sectional diameter of the apple in the image was W_p_ = 2R_max_.

**Fig 7:**
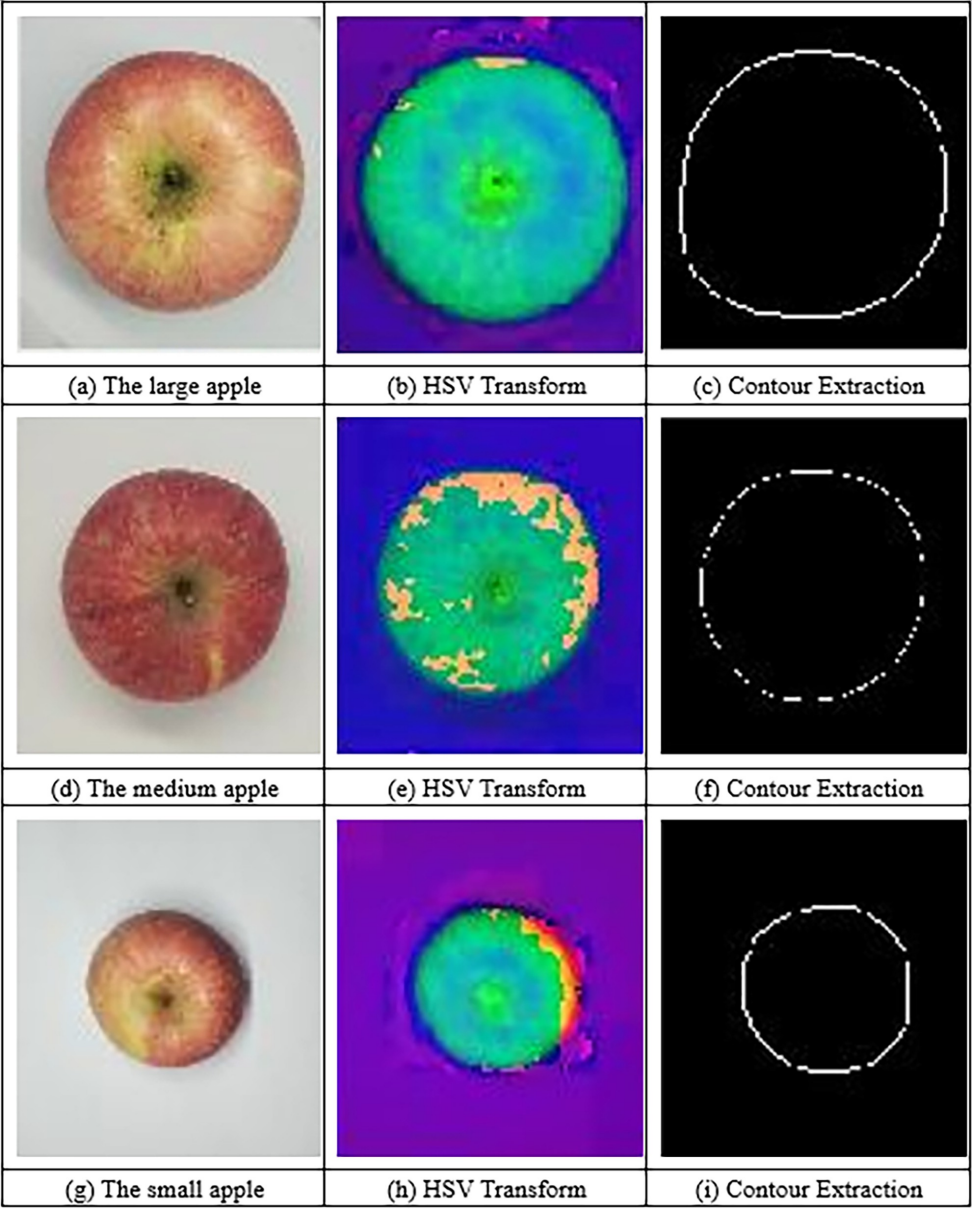
The extraction process of contours of apples with different sizes. (a) The large apple (b) HSV transform of the large apple (c) Contour extraction of the large apple (d) The medium apple (e) HSV transform of the medium apple (f) Contour extraction of the medium apple (g) The small apple (h) HSV transform of the small apple (i) Contour extraction of the small apple.

**Fig 8:**
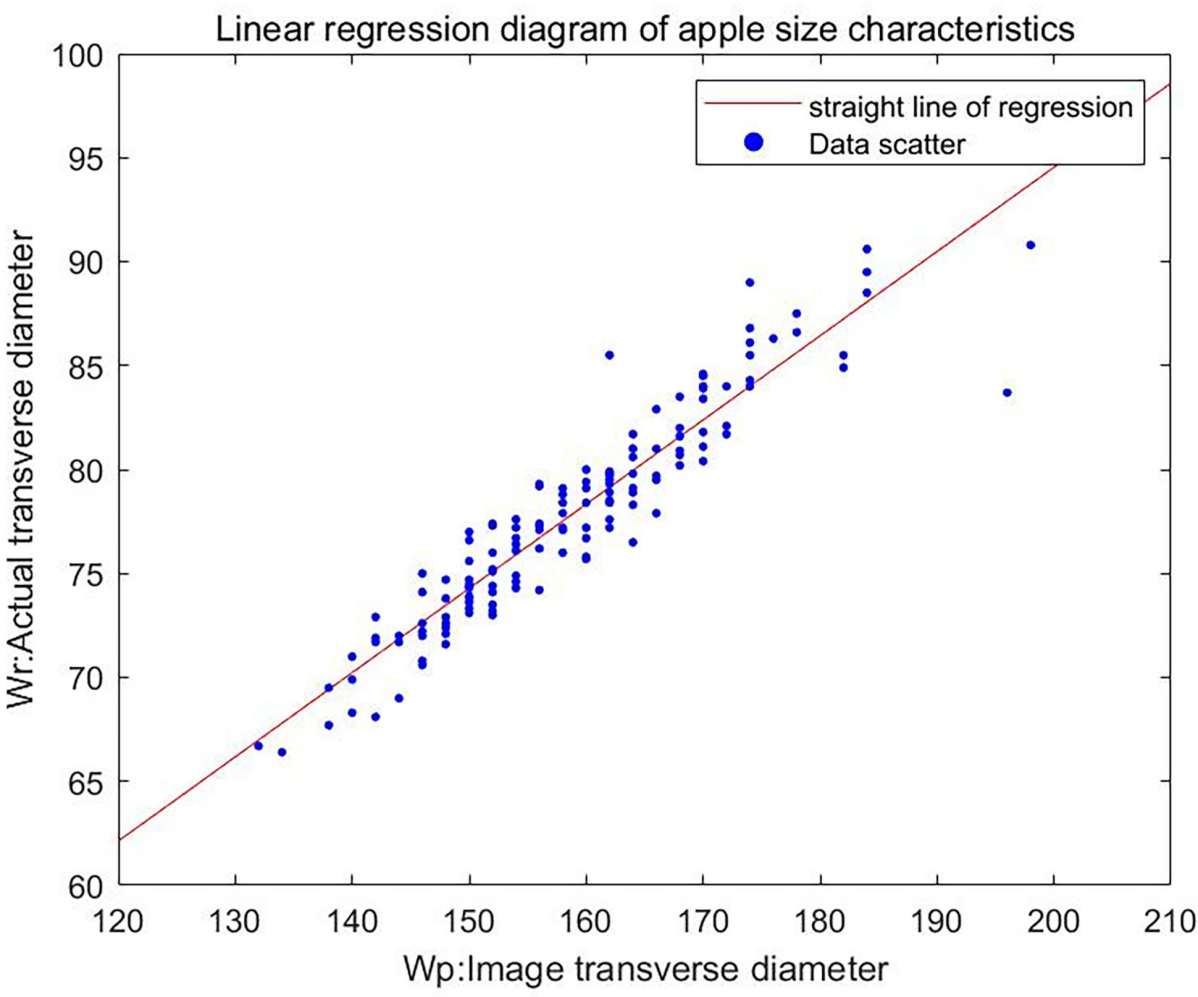
Linear regression plot of the apple size characteristic.

We selected 150 apple samples and used a vernier caliper to measure and record their maximum cross-sectional diameter W_r_. Then, we took photos and carried out the image processing shown in Fig 7. The maximum outer circle diameter of the apple image was obtained based on the minimum convex hull method, and it was taken as the maximum cross-sectional diameter W_p_ of the apple image. Thus, a linear regression model between W_r_ and W_p_ was constructed. The results are shown in Fig 8, and the regression equation is

$$W_{r}=0.4052W_{p}+13.5015$$ (7)

where the correlation coefficient *R*^2^ = 0.8462, the residual variance *S* = 3.9806, and the test of significance of variance *p*<0.001, demonstrating that the linear regression model has a high degree of accuracy and can meet the experimental requirements.

According to the national standard of the People’s Republic of China, the thresholds of cross-sectional diameter Wr for distinguishing among extra-large, large, medium, and small apples are 8 cm, 7 cm, and 6.5 cm respectively. Substituting these values into Eq (7), it can be seen that the thresholds for apple size grading according to the maximum cross-sectional diameter Wp of the apple image are 164, 139, and 127 pixels respectively, based on which the classifier for apple size grading was constructed, as shown in Eq (8).


$$W_{p}=\{\begin{array}{l} \geq 164,extra-large\;apples\\ 139\leq W_{p},164,large\;apples\\ 127\leq W_{p},139,medium\;apples\\ <127,small\;apples \end{array}$$ (8)

Fifty extra-large apples, 50 large apples, 50 medium apples, and 50 small apples were obtained, photographed, and after image processing, the cross-sectional diameter W_p_ values of these apples in images were calculated separately and substituted into the classifier for apple size grading. The experiment was conducted in triplicate, and the results are shown in Table 3. Statistics show that the average accuracy of grading apples for extra-large, large, medium, and small apples according to this classifier was 94.67%, indicating that the model can be used for the grading detection of apple size feature extraction.

**Table 3:** Results of detection for apple sizes.

Testing index	Detection number	First	Second	Third
Extra-large apples	50	48	47	48
Large apples	50	46	48	49
Medium apples	50	46	49	47
Small apples	50	48	46	46
Error		12	10	10
Accuracy		94.00%	95.00%	95.00%

## 5 Apple Brix prediction model construction

### 5.1 Near-infrared spectral image acquisition device

**Fig 9:**
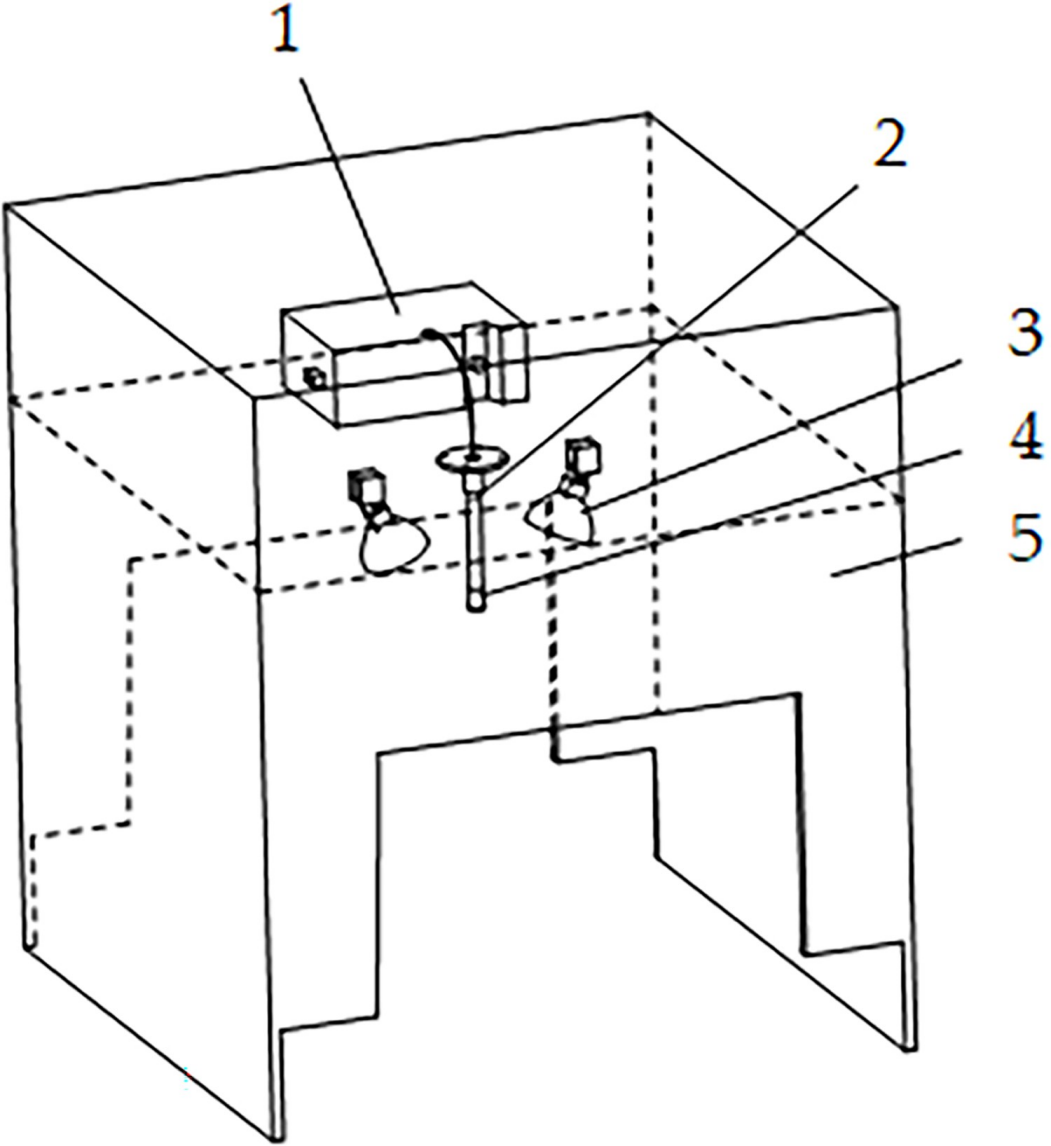
Sketch of the structure of the apple internal quality detection device. 1- Spectrometer 2- Fiber optic probe 3- Tungsten-halogen light source 4- Focusing lens 5- Dark box housing.

A near-infrared spectroscopy-based apple Brix detection device was constructed, as shown in Fig 9, mainly consisting of a spectrometer (USB2000+, Ocean Optics, USA), a tungsten-halogen light source (CH-20001, Changhui Electronic Technology Co., Ltd., Guangzhou), a fiber optic probe and probe holder (Changhui Electronic Technology Co., Ltd., Guangzhou), a focusing lens (Changhui Electronic Technology Co., Ltd., Guangzhou) and a dark box housing (PMMA). The acquisition device consists of two layers of the dark box structure. The upper layer was used to house the instrument and power supply and the lower layer detected the internal quality of the apples. The tungsten-halogen light source was arranged symmetrically at 45° on the bottom plate of the lower layer, and a 24V lithium battery (Laiyue Electronic Technology Co., Ltd., Guangzhou) was used to supply power to the tungsten-halogen light source.

### 5.2 Spectral curve acquisition and preprocessing

The surfaces of 168 apple samples were cleaned, and then the spectral curves of the apples were acquired by connecting the near-infrared spectrometer to a computer through a USB port and using spectral image acquisition software (Spectra Suite, 2020). The integration time of the fiber optic spectrometer was adjusted to 4 ms, the averaging time to 30, and the smoothing degree to 5. In this way, the final spectral reflectance curves of apple samples in the wavelength range of 400 nm~1000 nm were acquired. The black and white corrections were then carried out on the acquired curves according to Eq (9) to reduce the influence of the experimental environment on the results [29].


$$R=\frac{I-I_{d}}{I_{w}-I_{d}}$$ (9)

Where: *R—*the spectral reflectance of the sample, %;

*I—*the intensity of the reflection spectrum of the sample, cd;

*I*_*w*_*—*the reflected spectral intensity of a standard whiteboard (reflectance of approximately 95%), cd; and

*I*_*d*_*—*Reflected spectral intensity in the dark, cd.

**Fig 10:**
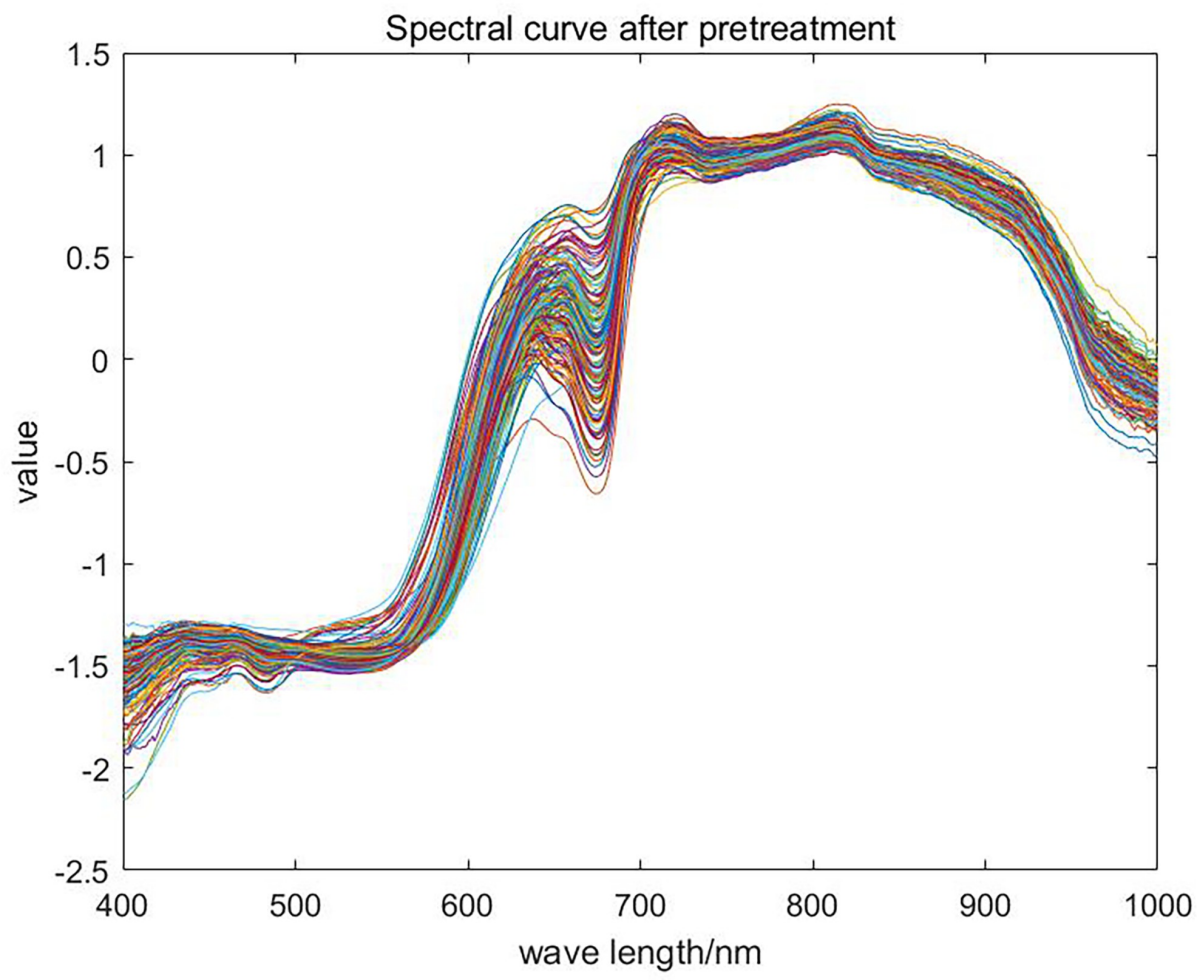
Spectral curves of samples after preprocessing.

A total of 1771 points were collected in the wavelength range between 400 nm and 1000 nm. To avoid additional noise interference, one wavelength point was selected every two points, and 591 wavelength points were finally selected. The spectral reflectance data of 591 wavelength points of the apple samples were used as the final data for the next step of processing. The multiple scattering correction (MSC) and standard normal variable (SNV) transformation methods were used to preprocess [30] spectral reflectance data of 168 samples to reduce the influence exerted by extraneous factors such as noise, equipment, and the experimental environment. The results are shown in Fig 10.

### 5.3 Apple Brix measurement

After obtaining the spectral data of the apples, the true value of apple Brix was measured using a handheld digital sugar meter (PAL-1 digital sugar meter, ATAGO, Japan). Each apple was peeled, diced, and mashed, and the juice was extracted to measure the apple Brix value. After each use of the sugar meter, the sampling tank was cleaned, rezeroed, and calibrated with water and then wiped clean with a dry paper towel, followed by the next measurement. The degree Brix values of 168 apple samples, as shown in Table 4, ranged from 8.9% to 14.6%, with a sample mean of 11.7%. Analysis of the experimental statistics showed that 90% of the apple samples had a Brix of 10% and above, and 15% of the apple samples had a Brix of 13% and above. Therefore, Brix values of 10% and 13% were selected as the thresholds for grading and dividing the Red Fuji apples in Luochuan, Shaanxi Province, into high, medium, and low grades based on their Brix values, with the grading criteria shown in Eq (10).


$$Apple\;Brix=\{\begin{array}{l} \geq 13\%,high\\ 10\%\leq Brix<13\%,medium\\ <10\%,low \end{array}$$ (10)

**Table 4:** Statistics on apple Brix.

Sample Number	Minimum	Maximum	Average	Standard Deviation
168	8.9	14.6	11.7	1.1

### 5.4 Characteristic wavelengths extraction

**Fig 11:**
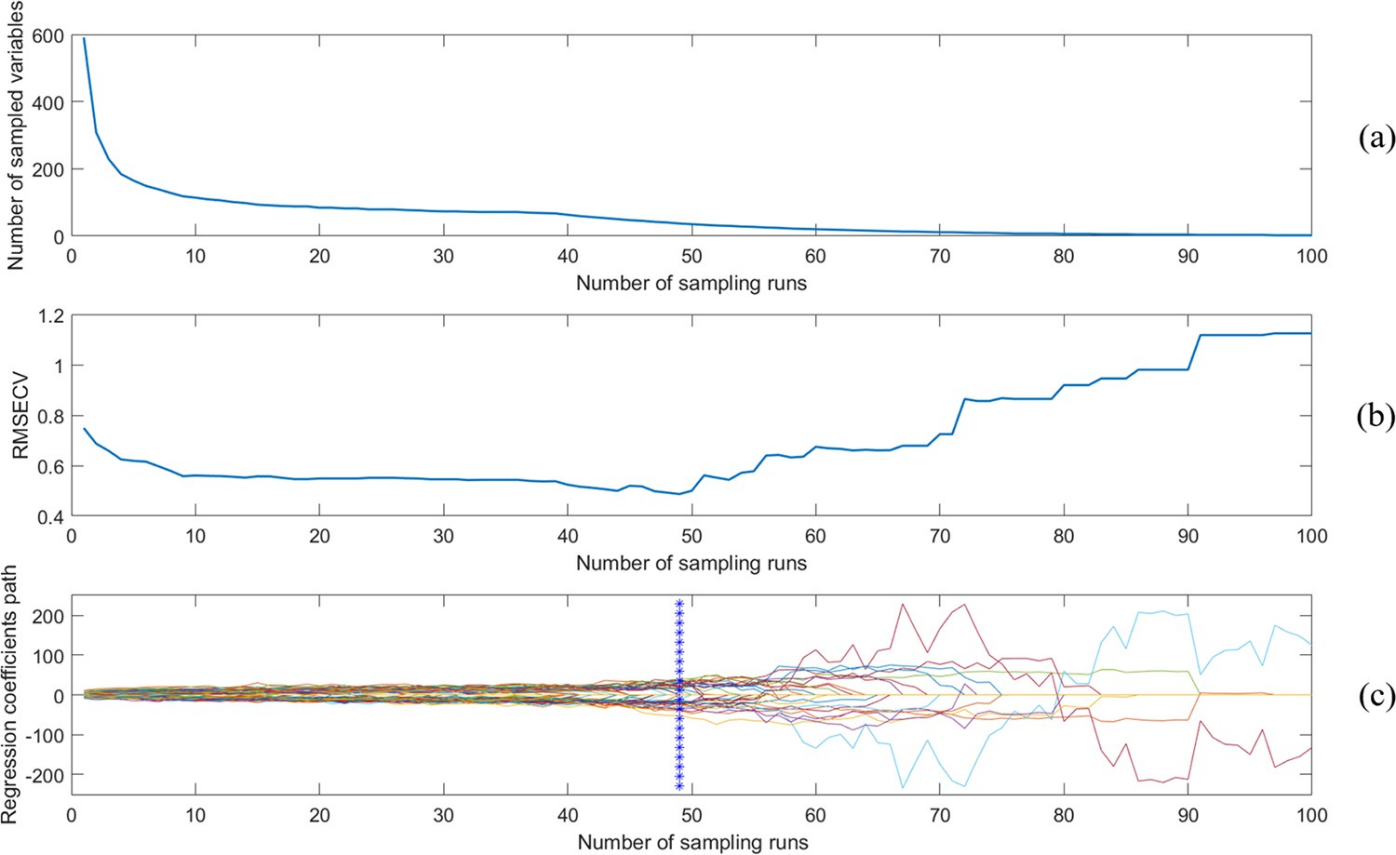
Graph of CARS characteristic wavelengths selection results.

The competitive adaptive reweighted sampling (CARS) algorithm and the successive projections algorithm (SPA) were used to filter the characteristic wavelength points related to the Brix to eliminate the irrelevant spectral data, reduce the computational effort and reduce the modeling time [31–33]. Fig 11 shows the results of the characteristic wavelengths selection using CARS, (a) the variation in the number of variables when different sampling times were selected, (b) the variation in the root mean square of cross-validation with the number of sampling times, and (c) a graph of the selection results of characteristic wavelengths using CARS. When the number of sampling times is 49 and the number of retained wavelength variables is 37, RMSECV reached a minimum value of 0.4869.

**Fig 12:**
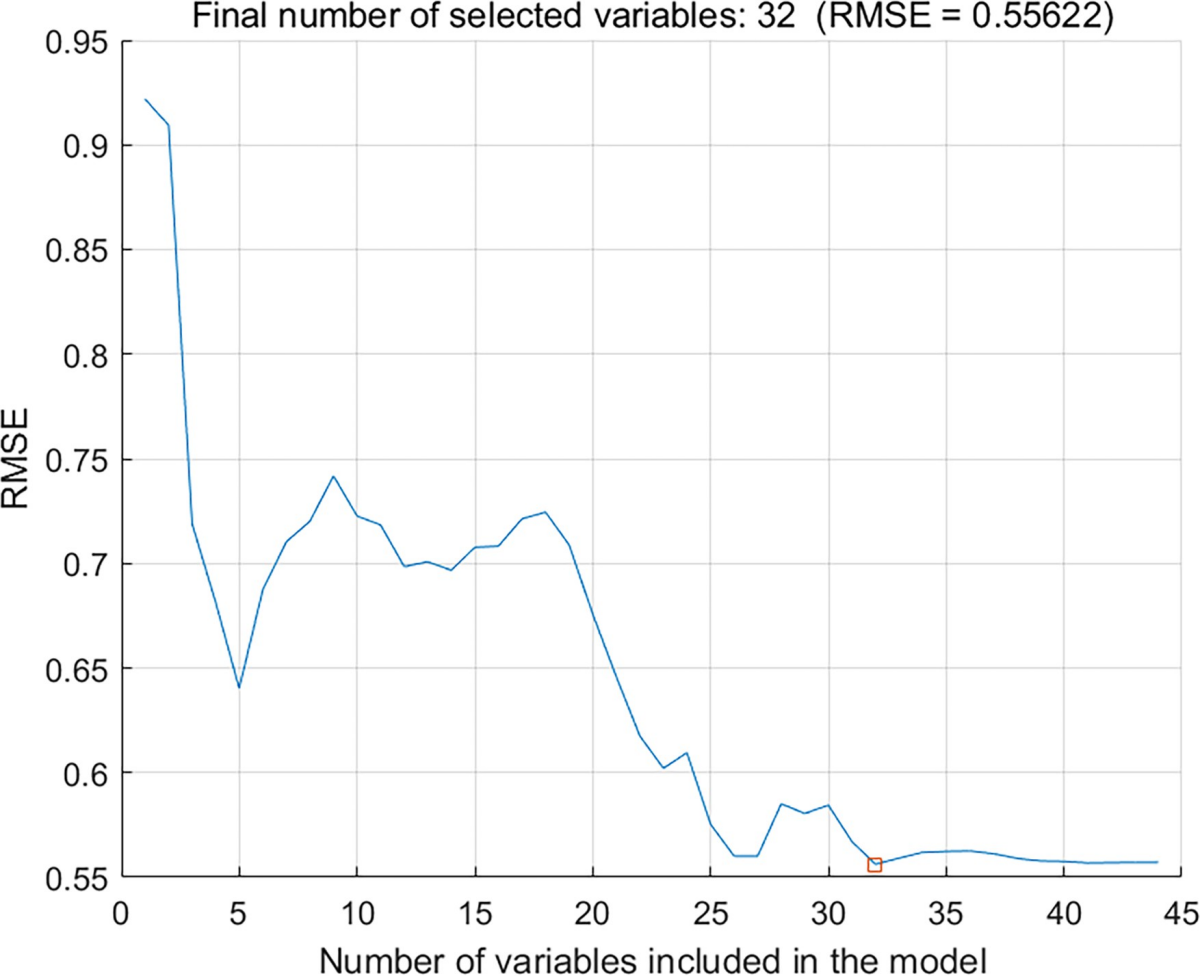
Number of SPA extracted characteristic wavelengths vs. RMSE curve.

**Fig 13:**
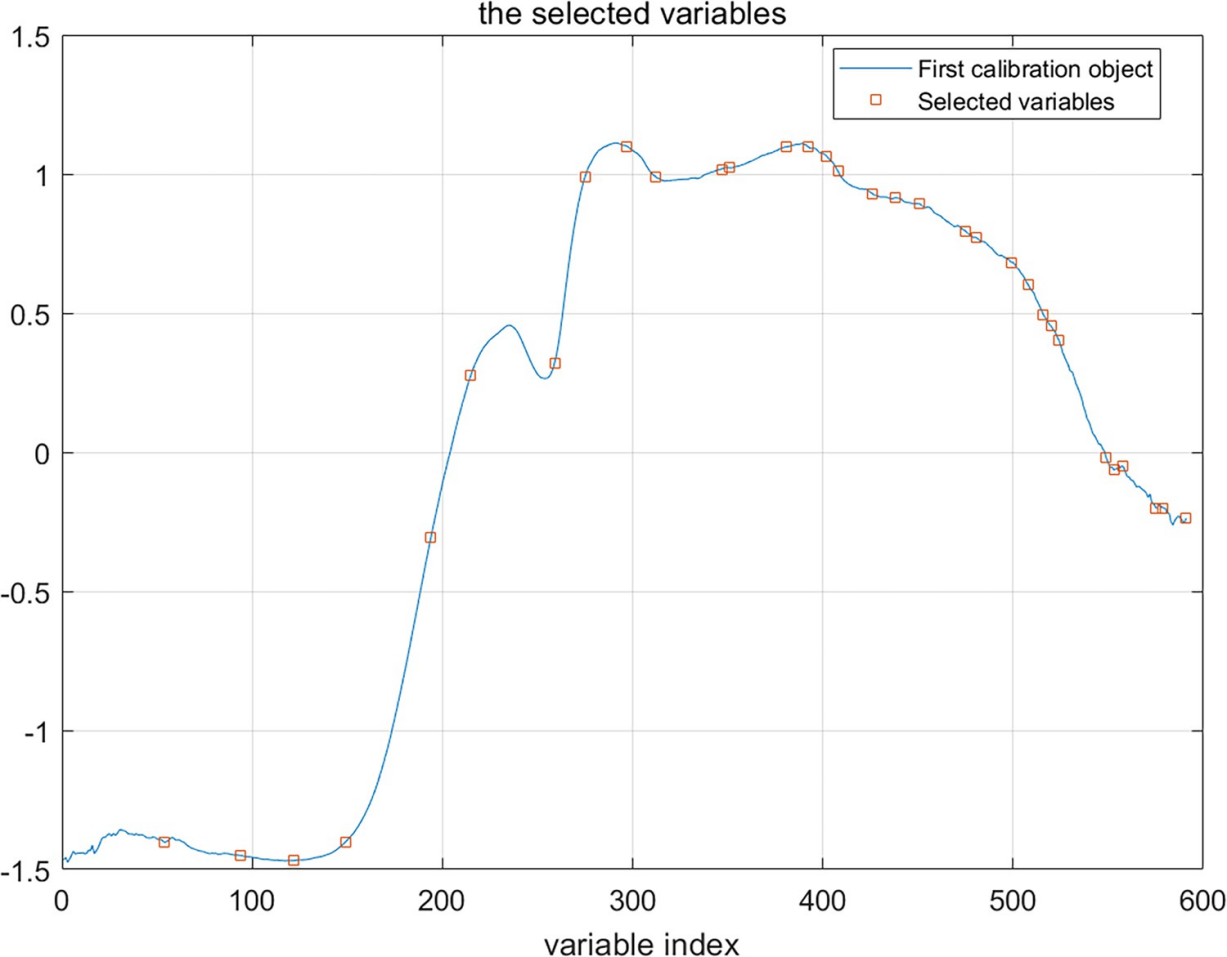
Map of SPA extracted characteristic wavelengths.

The range of the number of characteristic wavelengths was set from 30 to 50, the SPA algorithm was used to extract the characteristic wavelengths, and the number of wavelengths corresponding to the smallest RMSE was analyzed and calculated to obtain the optimal number of wavelengths, as shown in Fig 12, a graph of the number of characteristic wavelengths extracted by SPA and the RMSE. When the SPA algorithm extracted 32 characteristic wavelengths, as shown in Fig 13, the RMSE reached a minimum value of 0.5562.

### 5.5 Brix prediction model construction

Using the sample set partitioning based on joint x-y distance (SPXY) algorithm, 168 samples were divided into a validation set and a prediction set at a ratio of 3:1, with 126 samples in the former and 42 in the latter. The SPXY algorithm can achieve a uniform distribution space to ensure the validation set of samples [34], as shown in Eq (11)

$$d_{xy}(p,q)=\frac{d_{x}(p,q)}{\max_{p,q\in [1,N]}dx(p,q)}+\frac{d_{y}(p,q)}{\max_{p,q\in [1,N]}d_{y}(p,q)}p,q\in [1,N]$$ (11)

**Fig 14:**
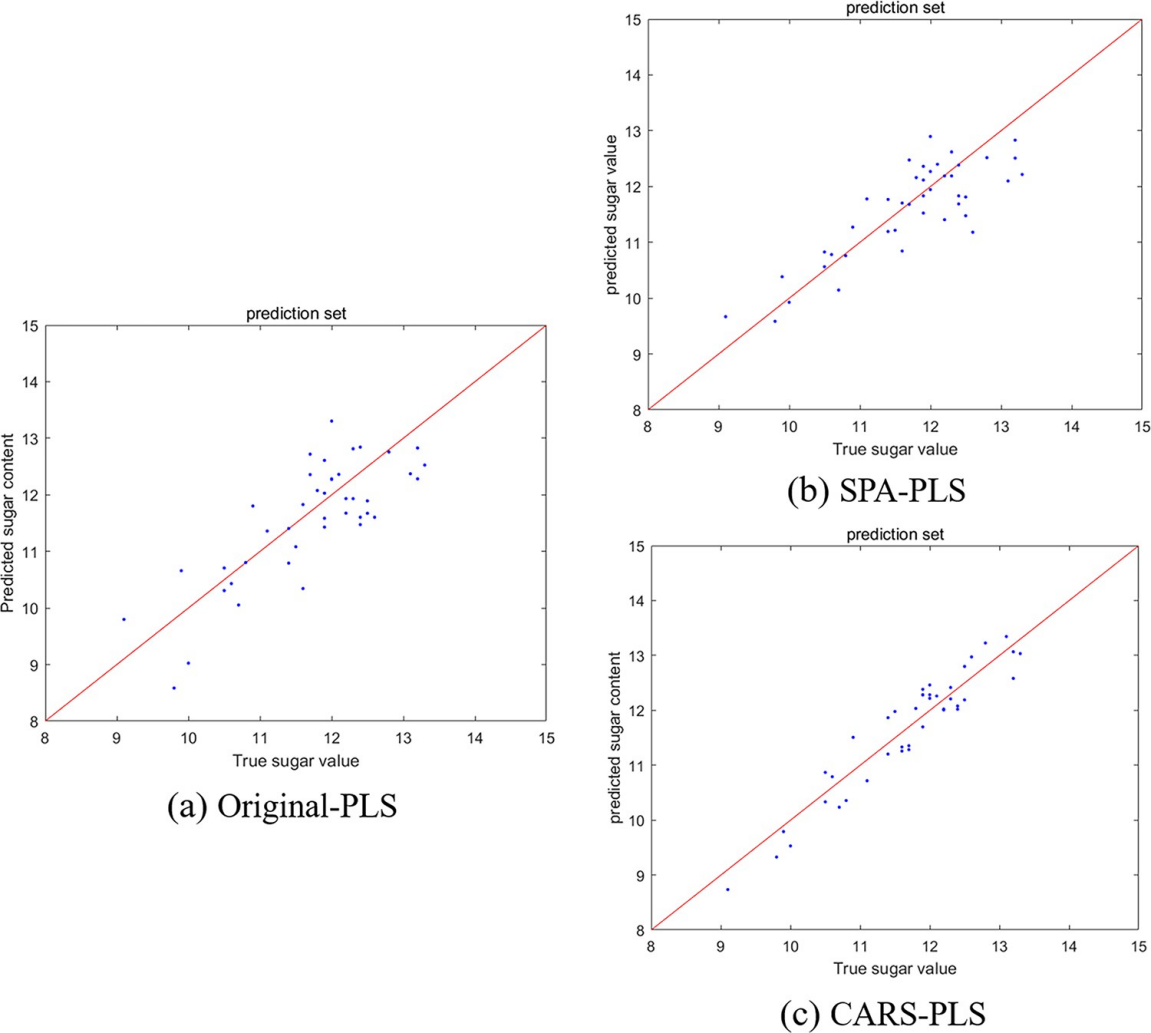
Scatter plots of the prediction results (in ° Brix). a. Original-PLS b. SPA-PLS c. CARS-PLS.

The performances of the three models were compared and analyzed as shown in Table 5, using the PLS algorithm [35] on the raw spectra, the spectra extracted by the SPA algorithm, and those extracted by the CARS algorithm. The comparative analysis of performances is shown in Table 5. After modeling with the PLS algorithm, the correlation coefficient of the prediction set R_p_ = 0.6543 for the original spectral data, and the root mean square error RMSEP = 0.7976, which indicated a large deviation. For the prediction model by extracting characteristic wavelengths using the SPA algorithm, the correlation coefficient of the prediction set R_p_ increased by 0.1787, and the RMSEP decreased by 0.2503. The correlation coefficient R_p_ of the prediction set obtained by extracting characteristic wavelengths using the CARS algorithm increased by 0.2926, and RMSEP decreased by 0.4461. The scatter plot of the predicted Brix results for the prediction set is shown in Fig 14, with the horizontal coordinates representing the true Brix values and the vertical coordinates representing the predicted Brix values. The better the fit of the scatter plot to the straight line *y* = *x*, the more reliable the predictions are and the closer the predicted values are to the real values.

**Table 5:** Comparative analysis of PLS model performances.

Algorithm	Characteristic wavelength number	Optimum PCs	R_c_	RMSEC	R_p_	RMSEP
Original-PLS	591	15	0.9227	0.4486	0.6543	0.7976
SPA-PLS	32	10	0.8680	0.5779	0.8330	0.5473
CARS-PLS	37	14	0.9563	0.3403	0.9469	0.3515

Comparing the four model evaluation index parameters, namely the correction set correlation coefficient R_c_, the correction set root mean square error RMSEC, the prediction set correlation coefficient R_p_ and the prediction set root mean square error RMSEP, it can be concluded that the Brix prediction model built using the CARS-PLS algorithm has optimal validation and prediction performance. A comparison of the three scatter plots of the Brix prediction results of the prediction set also yielded the same conclusion.

## 6 Experimental validation

### 6.1 Integrated apple grader design

**Fig 15:**
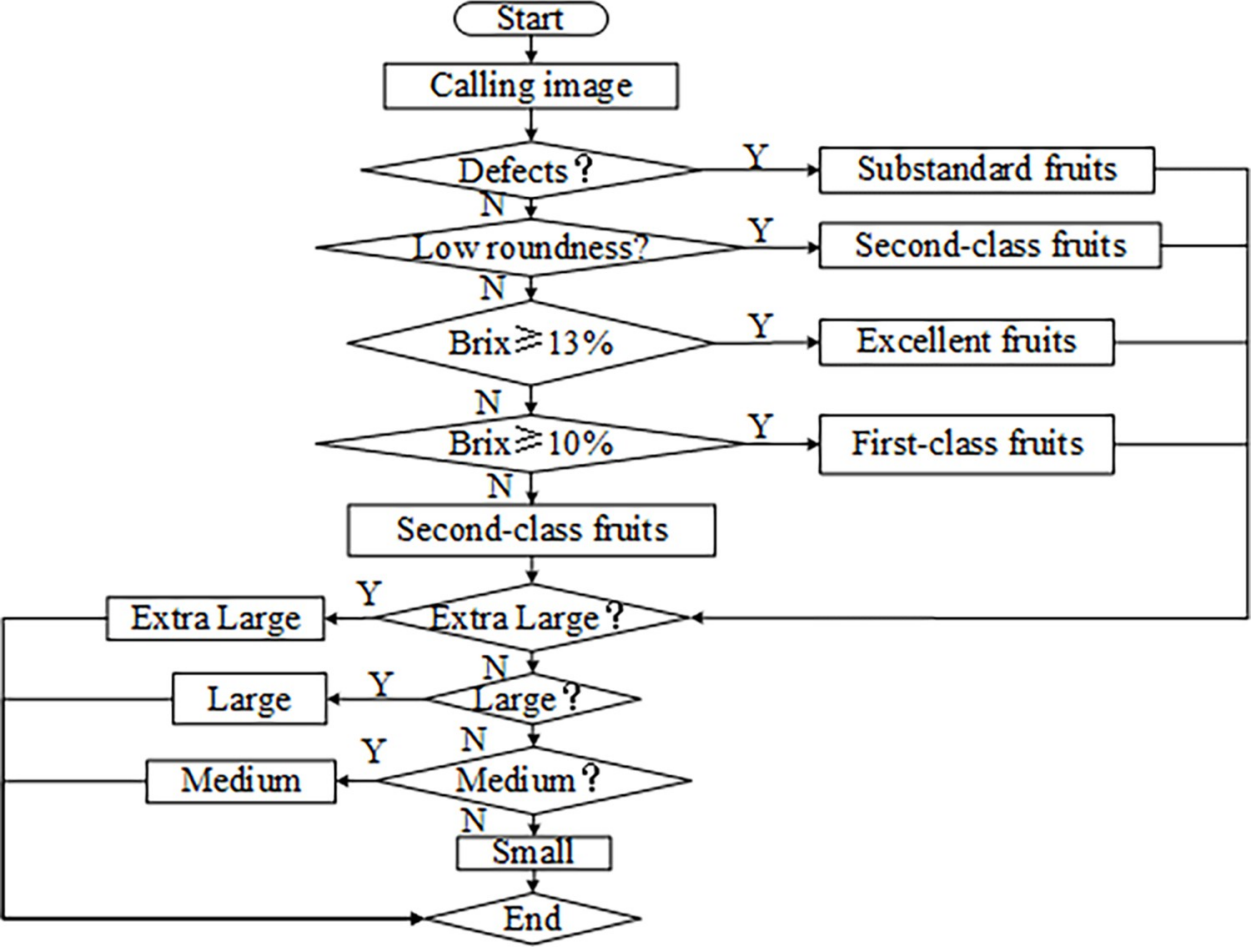
Flow chart of the apple quality grading algorithm.

Based on the above analysis, the apple grade was determined comprehensively based on different characteristics in terms of defects, shape, size, and Brix of the apple fruit. The algorithm flow of the apple grading is shown in Fig 15. First, the defects and shape of the apples are extracted to evaluate whether they meet the requirements. As long as defects exist, the apples are evaluated as substandard fruits. Among the apples meeting the requirements, those with high Brix values are evaluated as excellent fruits, those with medium Brix values are the first-class fruits, and those with low Brix values are second-class fruits; those with no defects but average shape are directly evaluated as the second-class fruits. Finally, the size features of the apple images are extracted to achieve the grading of extra-large, large, medium, and small apples.

### 6.2 Setting up the experimental platform

**Fig 16:**
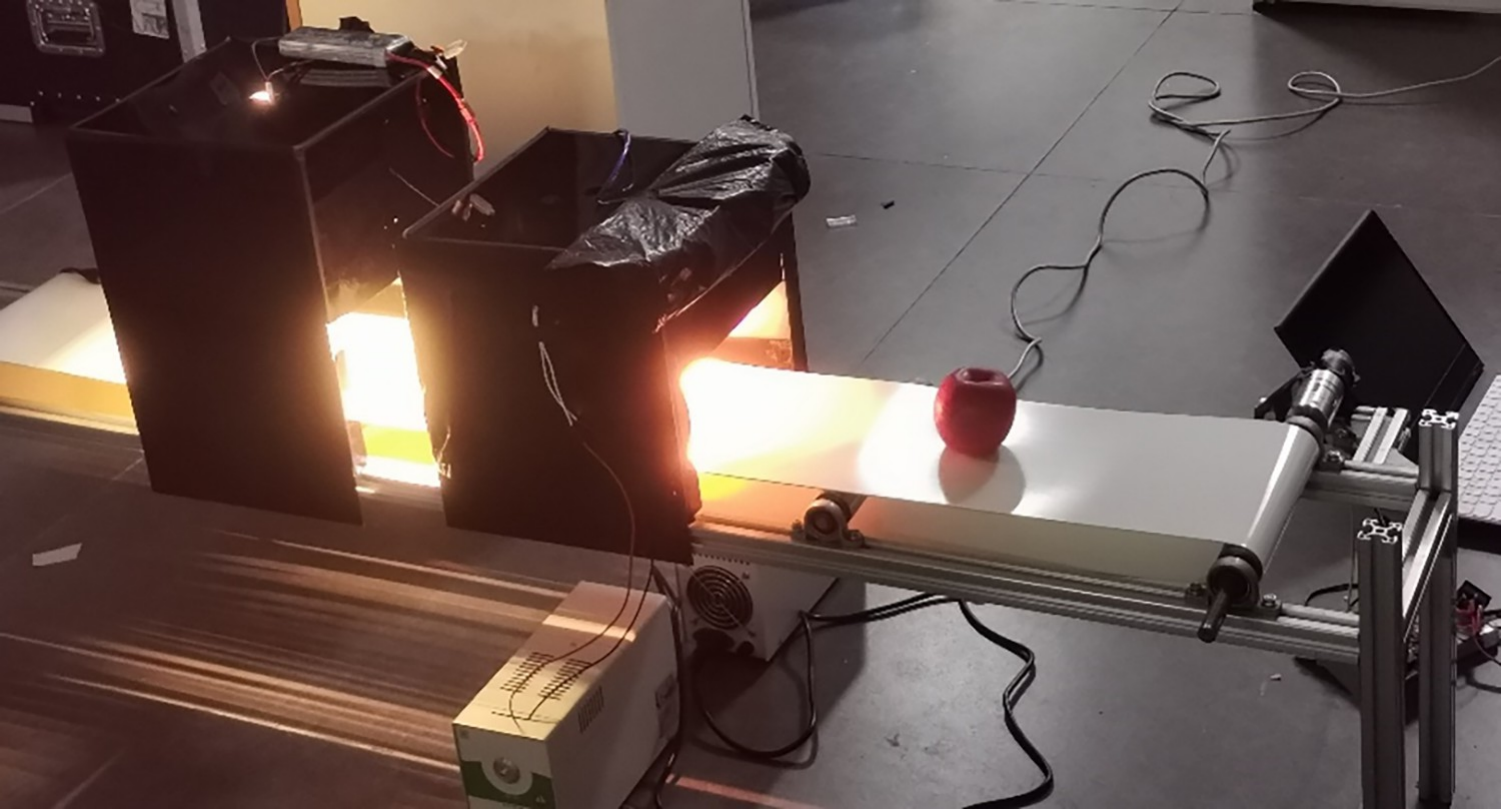
The prototype of the apple quality detection system.

To verify the feasibility of the grading algorithm, the prototype apple quality detection system shown in Fig 16 was set up. When an apple passes on the conveyor mechanism, a position detection photoelectric sensor in the vision-based detection box sends a low level to the PC device, which triggers the camera to capture the apple image. The device mainly realizes the detection of the parameters of defects, contour, and size of the apples. After the detection of appearance quality, the apples are sent to the internal quality detection system, and the collected spectral information is brought into the Brix prediction model to obtain the predicted values of the Brix. Finally, the developed upper computer software reflects the internal and external quality information and the corresponding parameters on the interface and inputs the detected parameters into the classifier models to assess the comprehensive grading of the apples and display it on the interface.

**Fig 17:**
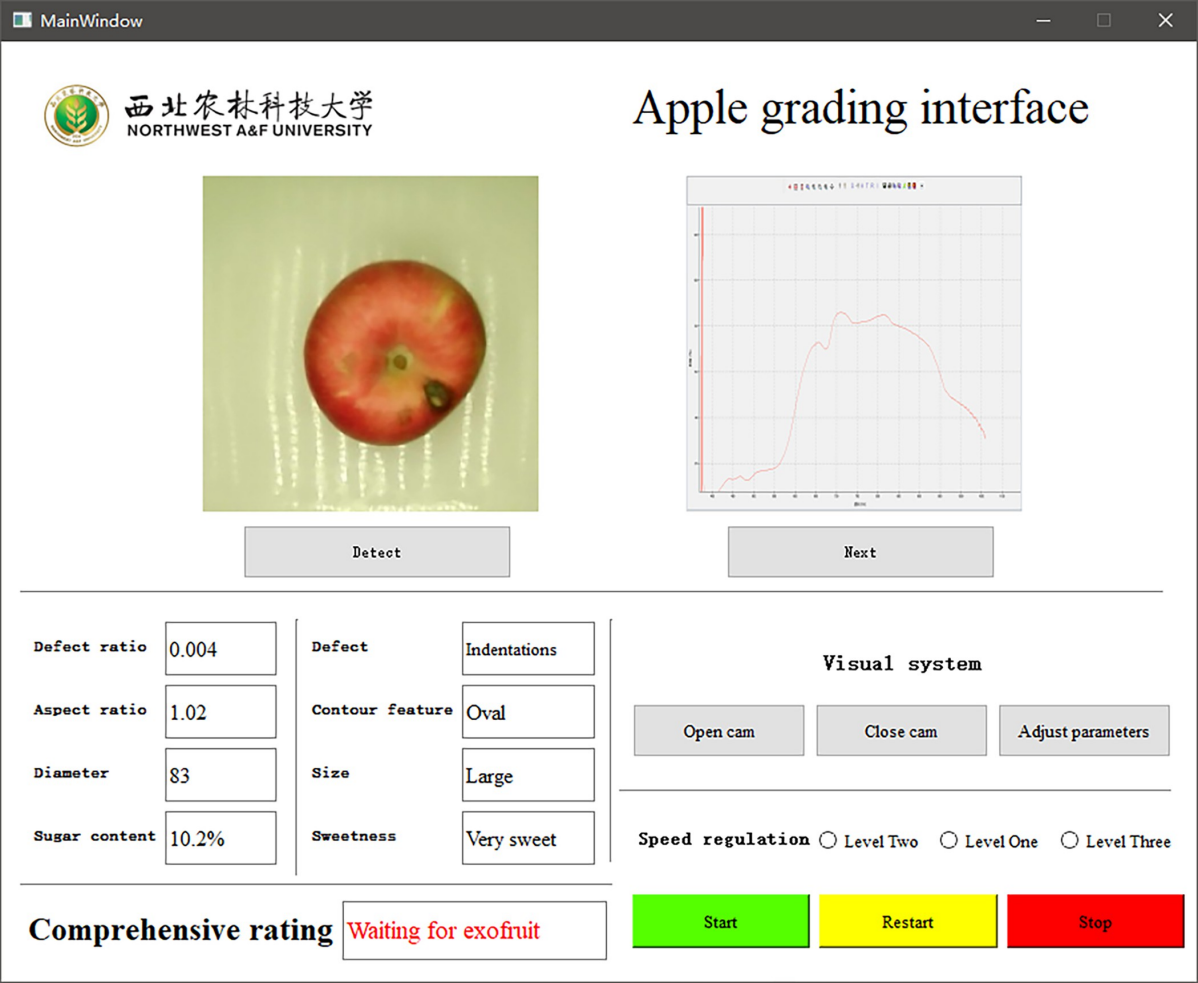
The interface of the apple quality grading software.

PyCharm Community Edition 2020 software was used to write the upper computer software under Windows, and PyQt5, a module of Python, was used to create the interface for apple grading detection. The interface for running this software is shown in Fig 17.

### 6.3 Experimental results

A total of 120 apples were randomly selected to validate the grading system and the results of the apple grading were output on the interface of the upper computer software. To allow for optimal image acquisition with the camera, the calyx side of the apple was uniformly placed towards the conveyor belt, and the stalk side was placed towards the camera on top of the detection box. The experiment was conducted in triplicate, and the grading results are shown in Table 6. The accuracy of apple grading detection was 95.83%, 97.50%, and 96.67%, respectively, with an average accuracy of 96.67%.

**Table 6:** The results of apple quality grading according to the national standard.

Grade	Detection number	First	Second	Third
Excellent apples	21	19	19	20
First-class apples	35	33	35	34
Second-class apples	49	48	49	47
Substandard apples	15	15	14	15
Error		5	3	4
Accuracy		95.83%	97.50%	96.67%

## 7 Summary and discussion

This paper analysed the principles of apple defects, shape, size, and Brix detection and grading based on China’s current national standard. In this research, Red Fuji apples in Luochuan, Shaanxi Province, were used as the research object, the appearance quality classifier models of apple defects, shape, and size characteristics were established based on machine vision, and the prediction model of apple Brix value was constructed based on near-infrared spectroscopy. The results showed that the average accuracy of apple defects, shape, and size characteristics grading detection was 96.67%, 95.00%, and 94.67%, respectively. Among the three Brix prediction models, Original-PLS, SPA-PLS, and CARS-PLS, the CARS-PLS model had the best prediction performance, achieving the following performance merits: R_c_ = 0.9563, RMSEC = 0.3403, R_p_ = 0.9469 and RMSEP = 0.3515. The validation experiment of the grading detection platform showed that the average accuracy of the grading detection was 96.67%, indicating that the apple grading system designed in this paper was feasible to some extent.

The accuracy of apple grading with this method can be further improved by improving the sensitivity of the photoelectric sensor and the operational stability of the transmission platform, both of which can reduce the position shift when the image is taken so that the apple is centered in the middle of the photo and the defects, shape and size characteristics can be extracted more accurately. If the distance between the fibre optic probe and the apple can be automatically adjusted according to the apple size, or the sealing of the near-infrared spectroscopy inspection device can be improved to reduce the interference of natural light on the spectral data acquisition, the accuracy of the Brix prediction of the system can be further enhanced.

According to the research ideas in this paper, with improvements in some algorithms, the apple grading detection system constructed can also be applied to the internal and external quality detection and grading of other nearly round fruits and vegetables such as pears, peaches, and tomatoes.

## Supporting information

S1 File The list of the grading detection device. (DOCX)

S2 File Original data. (XLSX)

S3 File Funding information. (TXT)
