# Supplementary material for: Grading detection of “Red Fuji” apple in Luochuan based on machine vision and near-infrared spectroscopy
Source: PLoS One. 2022 Aug 4;17(8):e0271352. doi: 10.1371/journal.pone.0271352 (PMC9352003; doi:10.1371/journal.pone.0271352)
Supplement: S1 File — (DOCX) [file pone.0271352.s001.docx]

| **The list of the grading detection device** | | | | |
| --- | --- | --- | --- | --- |
| Order | Equipment | Source | Notes | Website |
| 1 | Apples | Yan’an Shenggu Industrial Co Ltd | Red Fuji apples in Luochuan | http://yasgsy.com/shuiguojidi/ |
| 2 | Ring light source | Shenzhen Chuanghui Technology Co Ltd | Model: 2835-120 | http://jd.hc23.com/company/173093.html |
| 3 | Camera | Shenzhen Jierui Micropass Electronic Technology Co Ltd | Model: HF899 | https://shop109199787.taobao.com/?spm=a230r.7195193.1997079397.2.777646beGPyoCh |
| 4 | Photoelectric switch | Shanghai Huchuang Industrial Co Ltd | Model: E3F-DS30C4 | https://www.ch-hcdq.com/ |
| 5 | PC | Shenzhen Huawei Technologies Co Ltd | Model: HUAWEI MateBook 14 | https://www.huawei.com/cn/ |
| 6 | Spectrometer | Ocean optics | Model: USB2000+ | http://www.oceanoptics.cn/ |
| 7 | Tungsten halogen light source | Guangzhou Changhui Electronic Technology Co Ltd | Model: CH-20001 | https://www.gzchanghui.com/ |
| 8 | Fibre optic probe and probe holder | Guangzhou Changhui Electronic Technology Co Ltd |  | https://www.gzchanghui.com/ |
| 9 | Focusing lens | Guangzhou Changhui Electronic Technology Co Ltd |  | https://www.gzchanghui.com/ |
| 10 | Lithium battery | Guangzhou Laiyue Electronic Technology Co Ltd | Model: 24V | http://daogou.shiguangyouju.com/shop.php?shopid=126557863 |
| 11 | Conveying mechanism | Shanghai Parson Transmission Machinery Co Ltd | The conveyor belt | http://www.waixie.cc/com/shhpass021/ |
